# Presentation and physical therapy management of upper cervical instability in patients with symptomatic generalized joint hypermobility: International expert consensus recommendations

**DOI:** 10.3389/fmed.2022.1072764

**Published:** 2023-01-18

**Authors:** Leslie N. Russek, Nancy P. Block, Elaine Byrne, Susan Chalela, Cliffton Chan, Mark Comerford, Nicole Frost, Sharon Hennessey, Ann McCarthy, Leslie L. Nicholson, Jason Parry, Jane Simmonds, Patricia J. Stott, Lucy Thomas, Julia Treleaven, Wendy Wagner, Alan Hakim

**Affiliations:** ^1^Department of Physical Therapy, Clarkson University, Potsdam, NY, United States; ^2^St. Lawrence Health System, Potsdam, NY, United States; ^3^Advanced Therapy Programs PT, San Jose, CA, United States; ^4^Central Health Physiotherapy, London, United Kingdom; ^5^The Chalela Physical Therapy Institute for EDS/CCI, Charleston, SC, United States; ^6^Department of Health Sciences, Faculty of Medicine, Health, and Human Sciences, Macquarie University, Sydney, NSW, Australia; ^7^Performance Rehab, Brisbane, QLD, Australia; ^8^Comera Movement Science, Bristol, United Kingdom; ^9^Flex-Ability Physio, Wollongong, NSW, Australia; ^10^Not Just Bendy Hypermobility Services, Brisbane, QLD, Australia; ^11^Faculty of Medicine and Health, The University of Sydney, Sydney, NSW, Australia; ^12^University College London Hospital Trust, London, United Kingdom; ^13^Faculty of Population Health Sciences, University College London, London, United Kingdom; ^14^Elevation Wellness, Arvada, CO, United States; ^15^Neck and Head Research Unit, School of Health and Rehabilitation Sciences, University of Queensland, Brisbane, QLD, Australia; ^16^Wendy4Therapy, Naperville, IL, United States; ^17^The Ehlers-Danlos Society, London, United Kingdom

**Keywords:** hypermobile Ehlers-Danlos syndrome, generalized joint hypermobility, upper cervical instability, craniocervical instability, atlantoaxial instability

## Abstract

Experts in symptomatic generalized joint hypermobility (S-GJH) agree that upper cervical instability (UCI) needs to be better recognized in S-GJH, which commonly presents in the clinic as generalized hypermobility spectrum disorder and hypermobile Ehlers-Danlos syndrome. While mild UCI may be common, it can still be impactful; though considerably less common, severe UCI can potentially be debilitating. UCI includes both atlanto-occipital and atlantoaxial instability. In the absence of research or published literature describing validated tests or prediction rules, it is not clear what signs and symptoms are most important for diagnosis of UCI. Similarly, healthcare providers lack agreed-upon ways to screen and classify different types or severity of UCI and how to manage UCI in this population. Consequently, recognition and management of UCI in this population has likely been inconsistent and not based on the knowledge and skills of the most experienced clinicians. The current work represents efforts of an international team of physical/physiotherapy clinicians and a S-GJH expert rheumatologist to develop expert consensus recommendations for screening, assessing, and managing patients with UCI associated with S-GJH. Hopefully these recommendations can improve overall recognition and care for this population by combining expertise from physical/physiotherapy clinicians and researchers spanning three continents. These recommendations may also stimulate more research into recognition and conservative care for this complex condition.

## Introduction

Upper cervical instability (UCI) can be a serious and debilitating consequence in people with symptomatic generalized joint hypermobility (S-GJH). UCI may involve C0–C1 and/or C1–C2 joints, resulting in atlanto-occipital/craniocervical instability (AOI/CCI) and/or atlantoaxial instability (AAI) (1–4). Signs and symptoms of AOI and AAI overlap and, while they can be differentiated radiologically (1, 5), it is not always possible to differentiate them in the physical therapy clinic. Although physical/physiotherapists are likely to treat these patients, there are no published guidelines for safe, conservative assessment or management of UCI in this population (1, 5, 6). Diagnosis based on history and physical exam can be challenging as presentation can be quite variable between patients and even in the same patient over time, ranging from mild discomfort to severe disability and objective neurological signs.

Upper cervical instability (UCI) can result in myelopathy, cranial nerve neuropathy, brainstem compression, vertebrobasilar artery compromise and compromised venous or cerebrospinal fluid outflow. Vertebrobasilar artery problems are likely associated with AAI, while cervical medullary syndrome, cranial nerve problems and cerebrospinal fluid obstruction are likely associated with AOI (1, 4, 7). Symptoms of UCI include: headaches, neck or facial pain, dizziness, vertigo, nausea, paresthesias, dyspnea, dysphonia, vision changes (blurred or tunnel vision, visual aura), hearing changes, dysphagia, choking, sleep apnea, memory deficits, and pre-syncopal episodes. Signs associated with UCI include: long-tract findings such as hyper-reflexia, positive Babinski and Hoffman’s signs, loss of abdominal reflex, dysdiadochokinesia, as well as bowel/bladder problems, gait/balance deficits, weakness of arms and legs, sleep apnea and syncopal episodes (1, 5, 8). Dysautonomia is more likely to be present and severe with UCI and cervical myelopathy (9, 10).

Mild UCI in S-GJH may be relatively common (52–66%); (10, 11) while severe UCI is uncommon (5%) (11), it can be debilitating (8). UCI is likely underdiagnosed in S-GJH (5, 11). UCI in S-GJH has been the topic of several recent publications discussing imaging studies and surgical management. This recent literature asserts that UCI in S-GJH is an important condition to recognize, and there is a need for consensus-based recommendations, practice guidelines and care pathways for patients (2, 5, 6, 12). None of the recent publications address physical therapy assessment and conservative management for this population. Recognizing UCI is important to determine what physical exam tests (13) and interventions are safe.

The current work uses S-GJH as a surrogate for generalized hypermobility spectrum disorders (G-HSD) and Hypermobile Ehlers-Danlos syndrome (hEDS) (14). The presence of GJH is easily identified, using any one of many available hypermobility scores listed in Table 1. Although the Beighton score is widely used, many have questioned its validity and recommended other scales (15–18). Using GJH assessment tools makes the current recommendations more accessible to clinicians who are not HSD/hEDS experts, and easier to use with patients who have not yet been formally diagnosed with HSD/hEDS. Also, the HSD/hEDS diagnostic criteria may evolve in coming years (14). Research documents that cervical mobility is correlated to overall joint mobility assessed using Beighton score, so GJH is associated with cervical hypermobility (19–21).

**TABLE 1:** Assessment of generalized joint hypermobility (17, 18).

Beighton score Carter Wilkinson scoring system Rotes-Querol scale/criteria Hospital del Mar scale/criteria Upper limb hypermobility assessment tool Lower limb assessment score 5-point questionnaire*

*Also called the “5-item questionnaire.”

Hypermobility, mechanical instability, and functional instability are related but distinct phenomena. “Hypermobility” refers to excessive physiological motion at a joint for a persons’ age, sex, and race. “Mechanical instability,” or laxity, refers to excessive accessory motion at a joint, sometimes leading to subluxation, giving way, or dislocation. “Functional instability” refers to the subjective experience that joints may sublux, give way, or cannot be trusted, and is due to insufficient neuromuscular control at the joint (17). S-GJH, therefore, refers to people who are both hypermobile and experience symptoms. Symptoms may be musculoskeletal or neurological due to functional instability, or may be due to other issues common in G-HSD or hEDS, including but not limited to: fatigue, gastrointestinal problems, orthostatic intolerance, postural orthostatic tachycardia syndrome (POTS), urogynecological problems, mast cell activation, anxiety or depression (17).

The current work had several goals: (1) To bring together an international group of physical therapists with expertise in conservative care of S-GJH/UCI to exchange knowledge and begin ongoing discussions about best practices, (2) To compile expert opinion to develop screening, classification and conservative management recommendations for UCI in adults with S-GJH, and (3) To identify urgent needs for future research to allow development of evidence-based guidelines for UCI in S-GJH. This work also provides case scenarios exemplifying how patients with low, moderate, and highly irritable presentations of UCI might be screened, tested, and conservatively managed.

## Materials and methods

### Structure of consensus process

A Nominal Group Technique was used over a 1-year period from 2021 to 2022. The process included individual team meetings, asynchronous communication within each team, and 10 “full” group meetings including representatives from each team, as well as several asynchronous Delphi-type consensus processes such as voting (e.g., on most important signs and symptoms) and ranking (e.g., interventions safe for different levels of irritability). The number of and structure of individual team meetings varied to meet the needs of each team.

### Consensus expert selection

Once the idea of an expert consensus recommendation was proposed, members of the Allied Health Working Group of the International Consortium on Ehlers-Danlos syndromes and hypermobility spectrum disorders and the Ehlers-Danlos Society invited physical therapy/physiotherapy leaders from key regions of EDS PT expertise: US, UK, and AU. One HSD/EDS rheumatologist also participated in developing the recommendations; two neurosurgeons provided feedback on the draft manuscript. This report will refer to the representatives from each country as “teams” and members of the whole international group as “participants.” Each team (US, UK, and AU) had a “team leader” who facilitated discussions among team members between full group meetings, and presented or delegated that team’s presentation during meetings with the full group.

### Process

Literature review was performed on an ongoing basis, as topics arose, and included: assessment of GJH, UCI signs and symptoms, diagnostic testing for UCI, identification of red and yellow flags, and assessment of irritability. A search of other consensus-based PT-based recommendations and guidelines also provided a variety of models to consider. Individual teams began by meeting to define UCI and identify key signs and symptoms, then to develop recommendations for screening and diagnosing patients with UCI. The full group meetings discussed and modified models and recommendations several times until all participants were satisfied with a model that could guide clinical decision-making. Once the format of the model was defined, a more structured process was used to prioritize and rank the finalized elements within each component of the model. Case examples (see Supplementary material) were selected to reflect the decision-making process described in this paper.

## Results

### The expert panel

Seventeen clinicians participated throughout the consensus process. All participants except one were physical therapists/physiotherapists (one physician helped facilitate the process). All participants except one were recognized experts in S-GJH; all had considerable experience treating and many specialized in treating UCI in G-HSD/hEDS; one participant was an expert in cervical instability but was relatively new to S-GJH. Clinicians had an average of 26.7 ± 10.5 years clinical experience, 14.8 ± 10.4 years of research experience and 14.2 ± 8.8 years of academic teaching experience. Participants had 13.1 ± 6.6 (minimum 2, maximum 25) years of experience treating S-GJH and 10.6 ± 6.2 (minimum 3, maximum 25) years of experience treating UCI.

### Screening and classification based on history and symptoms

**FIGURE 1:**
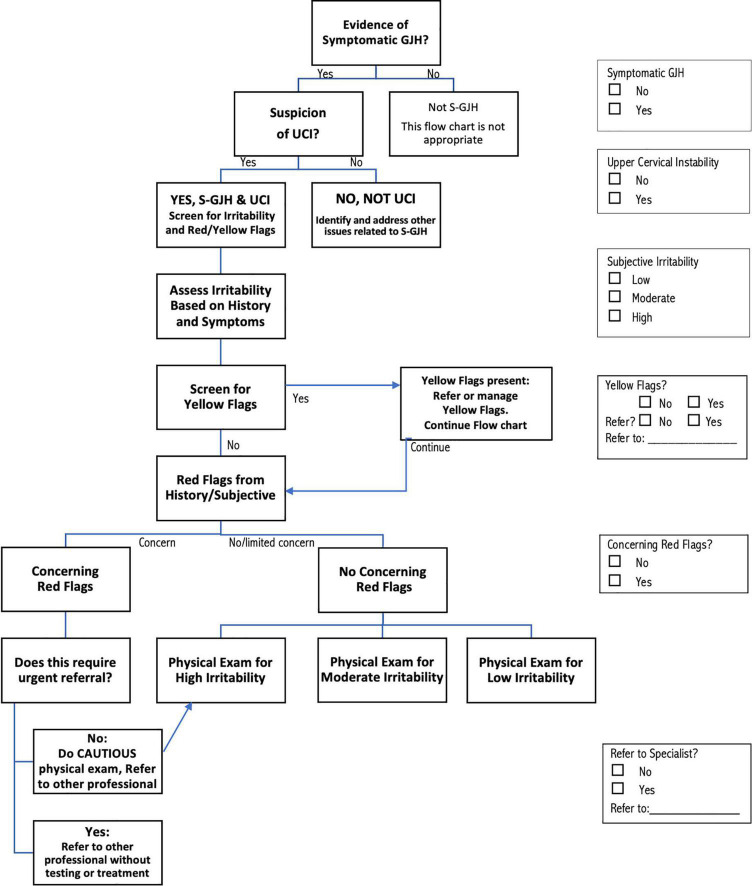
Flow chart describing the screening process for determining that patients have upper cervical instability (UCI) associated with symptomatic generalized joint hypermobility (S–GJH), irritability and identifying yellow flags and red flags. The screening steps can be implemented in any order, using the check boxes on the right to keep track of decisions made at each step. See text and tables for more detailed discussion of each step in the process.

**FIGURE 2:**
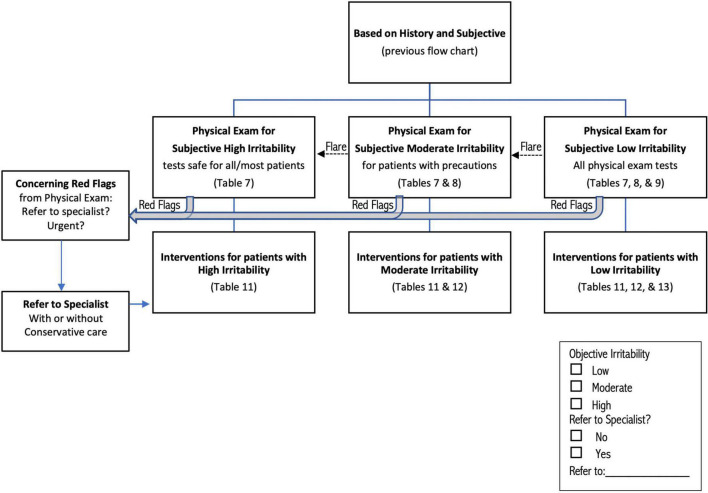
Flow chart describing classification of patients for physical examination and intervention, based on outcome of process described in Figure 1. Red flag physical test results (Table 6) can be identified in any group of patients; patients with red flags are automatically classified High Irritability. Irritability may increase during or up to 24 h after the physical exam (dotted arrow labeled “Flare”), shifting patients to a higher irritability group. See text and tables for more detailed discussion of each step in the process.

The recommendations for screening, assessment, and management of patients with S-GJH and UCI shown in Figures 1, 2 represent the consensus of the international participants. The screening and assessment process includes documenting evidence of S-GJH and UCI, as well as assessing irritability of the condition, and the presence of yellow or red flags (Figure 1). Except for assessment of GJH, this screening and assessment process is based on patient reported symptoms and history. Depending on the patient, some of this information may present naturally as patients describe their symptoms and history, while other information might need to be specifically drawn out in the interview. For some patients, asking about specific symptoms may increase their anxiety or result in confirmation bias. Clinicians need to use professional judgment regarding how the interview is performed. While the flow chart shows a proposed order for these steps, steps can be completed in any order; the check boxes on the right allow users to check off each step that occurs to ensure that all steps are in fact completed in whatever order is chosen.

#### Determination of symptomatic generalized joint hypermobility (S-GJH)

Determination of GJH can be done using any validated hypermobility test, with the current most common GJH assessments listed in Table 1 (17, 18). The Beighton score is practical for many clinicians: it is well-known, quick and easy to do and does not require tools if ranges clearly exceed the criterion. Other validated scales are also appropriate, especially if one has been previously used to assess GJH. The 5-Part Questionnaire (5PQ, also known as the 5-Item Questionnaire, 5IQ) may be particularly appropriate if physical assessment is not feasible, or if patients have lost range through age or injury (17). Symptoms may be musculoskeletal, neurological, or involving any of the other systems commonly affected in G-HSD/hEDS; patients seeking medical attention most likely meet the criterion of “symptomatic.” If the patient meets the criteria for S-GJH, clinicians can check the “Yes” box on the right side of Figure 1. Patients who present with GJH could be further assessed for G-HSD or hEDS (22–24) for more comprehensive management, but formal diagnosis of G-HSD/hEDS is not required. The recommendations proposed here are not intended for patients who do not meet the criteria for S-GJH.

#### Determination of upper cervical instability (UCI)

Determination of UCI requires that two criteria be met: (1) Symptoms consistent with musculoskeletal and/or neurological UCI and (2) Symptoms are altered by neck movement and/or position. Table 2 presents symptoms suggestive of musculoskeletal and neurological UCI. This information may arise naturally as patients describe their symptoms, or clinicians may ask about specific symptoms with the caution noted above about patient anxiety and confirmation bias. Patients do not need to report any specific number of these symptoms, as a few pronounced or consistent symptoms may be as informative as several milder symptoms. There is no research documenting sensitivity and specificity of these symptoms for UCI in S-GJH. However, clinicians working with these patients often have a sense of whether a symptom is more sensitive (most patients have it, but it is not specific enough to be diagnostic for UCI in S-GJH) or more specific (not all patients have it, but it is strongly suggestive of UCI in S-GJH when present). Therefore, Table 2 classifies the various symptoms as “common” or “highly suggestive” based on expert consensus until research documents sensitivity and specificity. “Highly suggestive” symptoms are less prevalent; therefore, the absence of these does not rule out UCI, especially in milder cases of UCI; however, the presence of these symptoms provides stronger evidence of UCI than for “common” symptoms. Similarly, the “common” symptoms do not individually provide strong evidence for UCI, though many common symptoms may create an overall impression of UCI. In contrast, the absence of any common symptoms should trigger consideration of other diagnoses.

**TABLE 2:** Symptoms suggestive of musculoskeletal or neurological upper cervical instability.

	Common	Highly suggestive
**Musculoskeletal UCI**		
● Heavy/bobble head, patient feels like they need to support or brace their head to decrease symptoms		**X**
● Apprehension about initiation or maintenance of neck movement or travel in vehicle		**X**
● Lump in throat, trouble swallowing		**X**
● Consistent clicking or clunking in the neck associated with neck movement		**X**
● Cervical sensorimotor symptoms such as tinnitus, dizziness		**X**
● Suboccipital headaches	**X**	
● Yoke/coat-hanger distribution pain	**X**	
● Neck tension, muscle spasm	**X**	
● Brain fog	**X**	
● Inconsistent or poor response to treatment for the neck	**X**	
● Sleep disturbance, snoring, sleep apnea	**X**	
**Neurological UCI**		
● Report of seizure-like activity, diagnosis of “non-epileptic seizures” or “pseudo seizures”		**X**
● Drop attacks not associated with dysautonomia (e.g., provoked by neck motion, or without dizziness common in POTS)		**X**
● Lump in throat, choking, trouble swallowing, voice changes		**X**
● Symptoms of dysautonomia (especially if not responding to standard treatment), persistent anxiety, functional GI dysfunction, poor temperature regulation, heat intolerance, presyncope,	**X**	**X**
● “Boat rocking” instability (not due to musculoskeletal issues)		**X**
● Ataxia: Poor coordination (not due to joint instability)		**X**
● Facial tingling/numbness		**X**
● Pulling sensation in face, head, teeth, tongue (muscle contraction, not just pain)		**X**
● Vision changes-trouble with convergence, double vision, aura (teichopsia)		**X**
● Dystonia: Involuntary muscle contractions causing involuntary movements or postures		**X**
● Intermittent dysesthesias in the limbs, not associated with local issues		**X**
● Sleep disturbance, snoring, sleep apnea	**X**	
● Cognitive changes		**X**

Upper cervical instability exists on a spectrum from mild forms causing discomfort but no hard neurological signs to more severe involving significant neurological compromise, with most patients having elements of both. Participants in the consensus process felt it was helpful to differentiate between musculoskeletal and neurological presentations, as musculoskeletal UCI is often milder and tends to respond better to conservative care than UCI with significant neurological involvement. UCI symptoms are quite variable among patients, and patients may demonstrate different features of UCI from day to day or within a single treatment as their conditions improve or flare.

The second criterion to determine high suspicion of UCI is that symptoms are altered by neck movement and/or position (Table 3). People with UCI typically report changing symptoms related to neck position or movement, or to activities and environments that stress the neck. Some patients will report increased symptoms with specific movements or postures, while others may be aggravated by any neck movement or perturbation. The conclusion that the patient has UCI is an overall professional impression based on the predominance of symptoms over time, and the link between these symptoms and provocation involving the neck. For example, a patient who loses consciousness when doing headstands as part of yoga practice might not have UCI while another patient who loses consciousness when sitting in a slouched posture might. Clinicians can check the appropriate UCI box (Y/N) in Figure 1. The case scenarios presented at the end of this article provide more examples of how information might be interpreted to determine high suspicion of UCI.

**TABLE 3:** Symptoms of upper cervical instability (UCI) are altered by neck movement and/or position.

● Increased symptoms with neck motion into, or when held in, flexion, extension, and/or rotation, especially increased neurological symptoms ● Apprehension about neck extension (e.g., washing hair, going to the hairdresser) ● Increased symptoms when leaning forward, looking down ● Increased symptoms with forward head posture, e.g., using computer keyboard ● Increased symptoms when upright with neck unsupported ● Decreased symptoms when in neutral or wearing a neck brace ● Apprehension, anxiety, or fear of manual exam to the neck

#### Determination that UCI symptoms are mechanically irritable

Mechanical irritability of UCI symptoms suggests that they have a mechanical cause (i.e., instability) rather than a systemic cause. Furthermore, assessing irritability is important to determine the safety of performing the physical examination or interventions to minimize flares (exacerbation of signs and symptoms). Table 4 lists the three components of mechanical irritability: (1) The condition is severe, (2) The condition is easily flared, and (3) Prolonged time to ease after flare. Patients may flare hours after the aggravating activity, so clinicians may need to reassess irritability when the patient returns and reports back. Irritability often varies over time, so the clinician needs to select the option most appropriate to the patient at this time. Irritability should be assessed for symptoms likely associated with UCI, and not based on other known problems, though this can be challenging in practice. Overall irritability can be graded using the criteria listed in Table 4, where the presence of more and more consistent components suggests higher irritability. The clinician can mark the appropriate box for irritability in Figure 1. In the current recommendations, irritability and red flags are used to identify what physical tests are likely to be safe and not likely to provoke a serious flare. Irritability can change over time, from day to day, and even within a single treatment session. Ideally, patients who improve will progress from higher to lower irritability.

**TABLE 4:** Symptoms are mechanically irritable.

**A. Condition is severe:** ● Poor tolerance to any time vertical ● Bed bound due to cervical symptoms ● Need to use a walker or wheelchair due to moderate or intermittently severe problems with coordination and balance rather than pain or weakness, or restricted to bed due to cervical symptoms ● Extreme cervical spine guarding with fear of movement secondary to severe reactivity ● Choking, trouble swallowing, and voice changes ● Profound visual disturbances ● Severe nausea with any neck movement ● Functional outcome measure relevant to UCI classified as Severe **B. Condition is easily flared:** ● UCI flares are disproportionate compared to provoking insult or activity. e.g., aggravated by minor rapid/unexpected movements/perturbations, traveling in car/bus, prolonged postures. ● Presyncope, syncope, drop attacks or seizure-like episodes with neck extension or rotation. ● History of excessive provocation associated with previous conservative care including hands-on manual therapy or exercise. **C. Prolonged time to calm after flare:** ● Provoked UCI symptoms take excessive time to settle to pre-flare state: e.g., more than 24 h for pain or more than several hours for neurological symptoms ● Pt regularly needs to resort to wearing a cervical collar or bedrest to ease symptoms after a flare ● Inability to tolerate being upright for > 24 h after flare **Grading mechanical irritability** ● Low irritability: ∘ A, B, and C are all typically absent, or ∘ B or C might be occasionally present at a low level. ● Moderate irritability: ∘ A, B, or C are intermittently present, or ∘ A or B or C is frequently present, but not all three consistently. ● High irritability: ∘ A, B, and C are all frequently present.

Clinicians should be cautious about interpreting pain severity in section A of Table 4, as multiple factors can influence pain severity. For example, overall pain may be due to comorbidities rather than UCI. Psychosocial factors also influence severity, and will be discussed with Yellow Flags, below. Severe symptoms may be present very briefly, such as momentary pain due to subluxations, while typical pain is milder. Finally, patients with nociplastic changes associated with pain sensitization are likely to experience more severe pain that might not be due entirely to mechanical causes. None of these factors invalidate the severity that patients experience but should be considered in assessing the mechanical irritability of UCI.

#### Screening for yellow flags

Yellow flags (YF) are psychosocial symptoms or risk factors that may exacerbate presentation of any medical condition, including UCI. Clinical practice guidelines consistently recommend screening for YF and it is particularly important in this population (25). YF often coexist with physiological dysfunction, and can often be managed concurrently, through providing psychologically informed physical therapy with or without referral to another healthcare professional (25–27). The presence of YF does not imply that signs and symptoms are not real, or that there is no physical basis for the signs and symptoms. The presence of YF simply indicates that there are psychosocial factors that should be addressed for optimal outcome (25, 26). For example, YF may result in higher levels of nociplastic pain (28), as well as psychosocial issues that may interfere with conservative care. Yellow flags should be addressed whether they are directly due to UCI or other issues the patient may be dealing with.

Research shows that PTs are not skilled at identifying YF based on general impression (29). Therefore the recommendations suggest using a screening tool. Any tool for assessing psychosocial issues could be used; Table 5 lists several that may be appropriate (30–35). The OSPRO-YF is a multidimensional YF assessment tool designed specifically for physical therapist use (30). The full Spider Impact Scale addresses a range of symptoms common in patients with S-GJH; the questions related to depression and anxiety are appropriate for screening YF (31, 32). Readers interested in learning more about screening and managing YF are referred to an excellent commentary by Stearns et al. (25). In Figure 1, the clinician can mark whether YF are present and, if so, whether referral is needed, and proceed to screening for red flags.

**TABLE 5:** Some recommended yellow flag assessment tools.

● OSPRO-YF 10 or OSPRO-YF 17: Multidimensional assessment tools for identifying YF in physical/physiotherapy patients (30) ● Anxiety and depression questions from the spider impact scale developed specifically for patients with HSD/hEDS (31, 32) ● Tampa scale of kinesiophobia (TSK) any version (anxiety and fear of movement) (33) ● Fear avoidance beliefs questionnaire (FABQ) (anxiety and fear of movement) (34) ● Orebro musculoskeletal pain questionnaires (multidimensional) (35)

#### Screening for UCI red flags based on history and symptoms

Red flags (RF) are indicators of potentially serious pathology that may require urgent follow-up with a specialist. The RF symptoms listed in Table 6 suggest severe UCI requiring specialist care. Any other RF, such as those for cancer, infection, etc., should also be noted, but are outside the scope of the current work. As with other aspects of these recommendations, patients may volunteer this information or clinicians may specifically request it, communicating with patients in a way that does not generate or increase anxiety. Clinicians need to use professional judgment in interpreting whether RF are concerning or can be explained in a benign manner. For example, there are many musculoskeletal reasons a patient with S-GJH may need to use a walker or wheelchair, so this might not be a concern; however, being unable to walk safely due to ataxia and pseudo seizures would be of concern. Similarly, long-standing brain-fog is common and often not an urgent concern, but inability to answer simple questions due to recent or sudden onset cognitive changes is a concern. As with many aspects of clinical care, interpretation depends on context. RF symptoms should be reconsidered in combination with physical exam findings (discussed below) to decide whether and to whom a patient should be referred. Referral may be to a specialist medical provider, such as a neurosurgeon, neurologist, cardiologist, physical therapist specializing in UCI and/or S-GJH, or to another appropriate provider. Clinicians can mark, in Figure 1, whether RF symptoms are concerning and if so whether and to whom referral should be made.

**TABLE 6:** Red flags (36, 42, 43).

**History and symptoms** ● Seizure-like activity, pseudo-seizures ● Rapidly progressing neurological signs with decreasing functional status ● Drop attacks or syncope not associated with orthostatic intolerance (e.g., HR and BP changes) ● Altered consciousness or memory, severe or frequent changes in cognitive status ● Increased bowel/bladder control dysfunction ● Headache worse with Valsalva maneuver ● Need to use a walker or wheelchair due to moderate or intermittently severe problems with coordination and balance rather than pain or weakness ● Symptoms significantly increased after MVA, whiplash, trauma **Physical examination** ● Abnormal central nervous system reflexes: Babinski, Hoffmann, clonus, hypertonia ● Abnormal cranial nerve findings: Altered visual field, eye movement, unequal pupil size, amblyopia (lazy eye), facial sensory loss ● Observed speech or swallowing dysfunction, choking, tongue dysfunction, sleep apnea (lower cranial nerves) ● Abnormal vertebrobasilar insufficiency tests with auditory and vision changes, evidence of vertigo, presyncope or syncope ● Ataxia, gross neurogenic gait abnormalities, inability to perform tandem gait, Romberg sign present ● Dysdiadochokinesia: e.g., rapidly alternating pronation/supination, grip release test, fast finger or foot tapping ● Dystonia, myoclonic jerking ● FASTER Indications of stroke: Face, Arms, Stability (standing), Talking, Eyes. R is for React.

After all stages of screening are complete, patients are classified as “Urgent referral without testing or treatment,” “High Irritability,” “Moderate Irritability,” and “Low Irritability” (Figure 1). Patients with concerning RF who are not directly referred are automatically included in the “High Irritability” group, which limits the physical exam to observation and neurological tests that do not involve neck motion or provocation, even if the patient met criteria for Low or Moderate Irritability.

Finucane et al. (36) present a model for interpreting spinal RF on a graded scale including “Emergency referral,” “Some concerning features,” “Few concerning features,” and “No concerning features.” These categories parallel our classification among Urgent Referral, High, Moderate, and Low Irritability. However, our recommendations use this classification to not only determine what intervention is likely to be safe, but also to guide what physical examination tests are likely to be safe. Finucane et al. (36) recommend creating a “safety net” for patients who might develop RF or whose RF may progress. “Safety netting” is a process where patients who are at risk of serious pathology are educated about what signs and symptoms to look for and what action to take if those signs and symptoms occur.

### Physical examination

#### Tests and measures

The physical examination, when deemed safe to perform, occurs after patients have been classified as “High Irritability” (including Concerning RF), “Moderate Irritability,” or “Low Irritability.” Figure 2 shows that patients may move to a higher irritability classification during the examination, either because symptoms flare and more indicators of irritability present, (Table 4) or because of test results, including RF signs, discussed below. Tests should be ordered by starting with those least likely to provoke symptoms, saving those most likely to provoke for the end, if still safe to perform.

A limited number of tests/observations are typically safe to perform on all patients (Table 7), while others can be performed carefully on Moderate and Low Irritability patients (Table 8), and a final group of tests is considered safe only for Low Irritability patients (Table 9). This means that only tests listed in Table 7 are recommended for patients with High Irritability (including concerning RF), while tests in Tables 7, 8 should be appropriate for patients with Moderate Irritability, and tests in Tables 7–9 should be safe for patients with Low Irritability. These recommendations are not absolute, as some High Irritability patients might tolerate additional tests, especially in the hands of clinicians with S-GJH/UCI expertise. On the other hand, patients classified as Low Irritability might not tolerate some tests. Note that the patient’s irritability level may change during testing, most likely increasing irritability due to tests provoking signs and symptoms; in this case, adjust testing by limiting tests for the higher irritability group.

**TABLE 7:** Physical test and findings, safe for all patients.

	Contributing*	Common*	Diagnostic*
**Observation based tests**			
Posture, full body, sitting and/or standing, and segmental alignment	X	X	
Breathing pattern (chest vs. diaphragmatic, excessive accessory muscle use)	X	X	
Significant muscle guarding or reluctance to move neck		X	
Observe gait for ataxia, gross, and fine motor dyscoordination not due to other joint hypermobility			X
Observe for cranial nerve VII dysfunction: Lip drooping, unequal smile, eyelid twitching			X
Observe for dystonia, myoclonic jerking			X
**Neurological tests**			
Cranial nerve III, IV, VI tests: Oculomotor nerve/eye movement			X
Reflex tests not involving neck: e.g., Hoffmann, Babinski, clonus			X
Cranial nerve X, XII tests: Uvula, tongue (avoid gag)			X
Dysdiadochokinesia: e.g., rapidly alternating pronation/supination, fast finger or foot tapping			X
Testing of hand dexterity (need to distinguish from finger hypermobility). E.g., grip release test			X
**Other tests**			
Palpation for muscle spasm, especially suboccipitals, sternocleidomastoid, levator scapulae, upper trapezius		X	
Use of a rigid cervical brace for several weeks decreases signs and symptoms			X

*Contributing factors = not diagnostic but providing information about potential causes.

*Common = findings that are likely to be fairly common, but not necessarily diagnostic.

*Diagnostic = findings that are likely to be less common, but more diagnostic.

**TABLE 8:** Physical tests and findings for moderate and low irritability patients only.

	Contributing*	Common*	Diagnostic*
**Other motion and control**			
Thoracic range of motion, range, and quality	X	X	
Scapular muscle strength and motor control	X	X	
Excessive use of temporomandibular muscles to provide cervical stabilization (secondary finding)		X	
**Neck motion and control**			
Cervical range of motion: Overall, looking for apprehension, range, and quality		X	X
Deep neck flexor recruitment efficiency	X	X	
Cervical stabilizer motor control inhibition and inefficient recruitment (e.g., craniocervical flexion test, suboccipital extensor test)	X	X	
Sensorimotor tests: Eye-head coordination, trunk-head coordination, smooth pursuit visual tracking	X	X	
Cervical proprioception: Joint position error	X	X	
**Other tests**			
Neurodynamic tests may be cautiously performed, eliminating or caution with neck motion	X	X	
Orthostatic intolerance: NASA lean test or stand test		X	X
**Structural tests**			
Cervical axial load in supine			X
Alignment of C1 (manual assessment)			X

*Contributing factors = not diagnostic but providing information about potential causes.

*Common = findings that are likely to be fairly common, but not necessarily diagnostic.

*Diagnostic = findings that are likely to be less common, but more diagnostic.

**TABLE 9:** Physical tests and findings only for low Irritability patients.

	Contributing*	Common*	Diagnostic*
**Ligamentous testing**			
Abnormal passive accessory intervertebral movements (PAIVMs) or passive physiological intervertebral movements (PPIVMs) at OA and AA (if trained)	X	X	
Alar ligament test			X
Modified sharp-purser cervical instability relocation test (NOT the provocation test)			X
Cervical distraction in supine		X	X
**Mobility tests**			
Isolated AA ROM		X	X
Neurodynamic tests with neck motion		X	X
**Provocation tests**			
Craniocervical flexion test provocation of UCI symptoms.		X	
Vertebrobasilar insufficiency positional test			X

*Contributing factors = not diagnostic but providing information about potential causes.

*Common = findings that are likely to be fairly common, but not necessarily diagnostic.

*Diagnostic = findings that are likely to be less common, but more diagnostic.

Tests safe for all patients, including those with High Irritability, focus on observation of things like posture, breathing pattern, and movement abnormalities such as ataxia and dystonia, as well as neurological tests that create minimal stress to the neck, such as Hoffmann and Babinski reflexes and some cranial nerve tests. Palpation of cervical muscles may or may not be tolerated; if in doubt, consensus opinion is that touching the cervical spine should be avoided.

Although sensitivity and specificity of physical examination tests for UCI in S-GJH are not known, participants in this process rated whether a given test or observed characteristic is, in their opinion, more sensitive/common or more specific/diagnostic, and this is noted in Tables 7–9. Additional tests relate to contributing factors that may need to be addressed to resolve the condition, and are generally not diagnostic. For example, forward head or the head tipping forward both place significant stress on structures in the upper cervical spine. Depressed or downwardly rotated scapulae suggest scapular instability and excessive strain on cervico-scapular muscles and fascia. A flattened mid-thoracic spine with rhomboid overactivity may suggest dural sensitization.

Motor control is particularly important for cervical stability, as instability is due to insufficient neuromuscular control and inappropriate recruitment patterns. Central nervous system inhibition of stabilizer synergist recruitment often persists long after pain flare ups resolve contributing to innocuous and insidious recurrence (37, 38). If small range cervical movement is deemed safe to do (Table 8), clinicians can assess whether the deep neck stabilizers are effective and efficient. For example, are the deep neck flexors able to generate small, controlled movements without recruitment of the sternocleidomastoid, scalenes, hyoids, and temporomandibular muscles? (37) Detailed discussion of motor control is beyond the scope of the current work, and readers are referred to the text by Comerford and Mottram (38).

#### Red flags in the physical examination

Figure 2 shows that the physical exam may identify RF signs (Table 6) which combine with RF symptoms to determine the need for referral to another professional and extra cautious intervention. RF signs include hard neurological or neurovascular findings consistent with UCI, such as cranial nerve pathology, vertebrobasilar insufficiency, or cervical myelopathy, as well as signs of non-UCI conditions such as stroke. Although High Irritability patients are most likely to demonstrate concerning RF, patients in any level of irritability may present with RF. Various criteria exist for surgical treatment, and have been reviewed; (5) one set of criteria are (1) moderate to severe headache or suboccipital pain; (2) bulbar symptoms indicating cervical medullary syndrome; (3) neurological findings indicating myelopathy, and (4) radiographic evidence of instability (8). The therapist may choose to refer the patient without any conservative care, or refer while providing cautious conservative care. As with RF symptoms, patients should be “safety netted” through education about signs of serious or emergent pathology and what action to take if those signs occur.

#### Evaluation, diagnosis, prognosis

After the patient interview and physical examination, the therapist performs an evaluation, and provides a diagnosis, and prognosis. The evaluation involves identification of contributing factors, especially those that can be addressed through conservative care. Contributing factors likely include excessive neck mobility, poor posture, sensorimotor and proprioceptive deficits, inappropriate motor recruitment of stabilizing muscles in the neck, dysfunctional breathing patterns, and inappropriate body mechanics (especially during activities of daily living).

The physical therapy diagnosis involves confirmation that signs and symptoms appear consistent with UCI, or are a different diagnosis, or both UCI and another diagnosis. Table 10 lists differential diagnoses that share signs and symptoms with UCI, and therefore need to be considered as alternatives or as co-morbidities (1, 6, 39, 40). Differentiating among these can be challenging, may require diagnostic imaging, and is beyond the scope of this article. The experts who participated in the current work generally agreed that there are different types of UCI. For example, anterior-posterior (AP) instability (probably due to either CCI or AAI) and rotational instability (probably due to AAI) might be distinguished through additional physical examination testing and interpretation of findings. However, to avoid futher complexity, these subtypes of UCI were not differentiated in the current model.

**TABLE 10:** Differential diagnoses that should be considered (1, 6, 39, 40).

● Chiari malformation ● Migraine/headache ● Intracranial hypotension (cerebrospinal fluid leak) ● Idiopathic intracranial hypertension ● Tethered cord ● Eagle syndrome ● Dysautonomia unrelated to cervical instability ● Functional neurologic disorder ● Functional movement disorder ● Tarlov cysts

If UCI is the primary diagnosis to be managed conservatively, the therapist reviews the findings to determine whether the patient’s irritability level should be changed from that based on the patient interview, given the results of the physical exam and any RF signs. The therapist should also reconsider the need for referral to address YF and/or concerning RF.

Prognosis depends on many factors, such as contributing factors, the presence of YF or nociplastic changes, and the initial severity. Primarily musculoskeletal UCI is often well-managed through appropriate conservative care, whereas neurological UCI appears to be more challenging.

### Interventions

#### Interventions appropriate for all patients

Interventions have been organized similar to tests and measures, with those interventions that should be safe and appropriate for all patients, those that should be safe and appropriate for patients with Moderate or Low irritability, and those safe for patients with Low Irritability. Patient classification as Low, Moderate, or High Irritability may change from day to day, and may progress toward lower irritability as patients improve. As with other aspects of these recommendation, therapists need to use professional judgment for both selecting interventions and monitoring their tolerance. Table 11 shows interventions that are considered safe and appropriate, even for High Irritability patients, as well as interventions to be avoided. Interventions for High Irritability patients focus on education about posture, body mechanics, and functional activities, as well as pain science and pain self-care. More detailed examples of functional training for patients with High Irritability are described in Supplementary Box 1. Relaxation techniques and autonomic nervous system balancing including, but not limited to, slow diaphragmatic breathing or heart-rate variability biofeedback may help decrease pain sensitization and allow more active engagement in physical therapy.

**TABLE 11:** Interventions for all patients, and interventions to avoid in high irritability patients.

**General education** ∘ About S-GJH and UCI ∘ “Safety netting”: recognizing signs and symptoms that trigger emergency or urgent follow-up or referral; self-care in these situations (e.g., wear cervical brace) **Posture and body mechanics education** ∘ Sitting, standing, and sleeping posture, positioning, and body support ∘ Body awareness and mindfulness in various positions (sitting, standing, lying down) ∘ Avoiding or limiting neck motion if small range motion is safe ∘ Functional training for posture and joint protection during essential ADLs such as bathing, brushing teeth, brushing hair, washing hair, sleeping postures, putting in contacts, eating, etc. ∘ Body mechanics, ergonomics, joint protection, activity pacing ∘ Orthotics and braces, as needed throughout the lower extremities and lumbar spine, to provide stable base for cervical spine ∘ Importance of shoe-wear support for spinal alignment **Pain science and pain self-care** ∘ Relaxation, autonomic nervous system balancing (not requiring neck movement) ∘ Breathing, e.g., diaphragmatic or slow breathing ∘ Pain neuroscience education, addressing catastrophization, mindful use of language to enhance feelings of safety ∘ Self-care “toolbox”: e.g., pain management strategies (e.g., heat, ice, transcutaneous electroneural stimulation, topical analgesics, relaxation, positive thinking, etc.) **Neck bracing (if appropriate)** ∘ Education about use of neck brace: how to put on, how often to use, when to use (e.g., during ADLs, flares, car travel) ∘ Custom fitting of rigid or soft cervical brace **Manual therapy** Some high irritability patients will not tolerate manual therapy, even remote from the neck, and it should be discontinued if not tolerated. ∘ Cautious myofascial release, trigger point release or neuromuscular inhibition techniques in the thoracic and lumbar spine, scapulae, lower and upper extremities. ∘ Cautious myofascial release, trigger point release or neuromuscular inhibition in the upper trapezius, levator scapulae, and sternocleidomastoid ONLY by clinicians with S-GJH/UCI expertise **Motor control** Some High Irritability patients will not tolerate motor control training, even remote from the neck, and this should be discontinued if not tolerated. These should be done with neck, torso and limbs suitably supported, generally in neutral position. ∘ Eye movement muscle energy techniques ∘ Pelvic and lumbar stability training; finding pelvic neutral. Ensure that the cervical spine is optimally aligned and supported ∘ Motor control training of the cervical spine, near mid-line ∘ Supine with head supported, scapular recruitment in neutral “safe zone,” side lying supported head and arm **Aerobic exercise** ● e.g., Recumbent bike, pedal exerciser (if there is no indication of neural tension/tethered cord) **Interventions to AVOID with high irritability patients** ● Exercises involving moderate to large neck movements, such as cervical range of motion ∘ Some patients will not tolerate any neck movement, even chin tucks ∘ Isometrics with more than minimal force ● Cervical axial loading (weight on head) or distraction (manual or mechanical) ● Only therapists with S-GJH/UCI expertise should perform any manual therapy to the cervical spine, and some patients may not tolerate any manual therapy, even by experts ● Positioning that creates neural tension (e.g., pelvic tilt in some people) or isometric load (e.g., quadruped) to the cervical spine

Patients in the High Irritability group may benefit from fitting and education regarding the use of neck braces, which can cue patients to maintain optimal cervical alignment. There are no published guidelines regarding use of neck braces in this population, and best use likely varies depending on the patient and context. While bracing has been controversial, the use of bracing can be empowering for patients avoiding social situations due to fear of neck flare up or injury. A brace can allow participation where this would not have been possible before. However, the use of bracing must be balanced with the potential complication of muscle wasting, which could worsen long term prognosis. For example, some patients should limit use of their neck brace to 15 min “rest breaks,” traveling (car, bus, etc.) or times of flare. On the other hand, patients waiting for a neurosurgical consultation might benefit from wearing a brace 24/7. A neurosurgeon specializing in S-GJH/UCI recommends patients perform gentle isometric resistance in the brace for a few minutes each day to maintain muscle tone^1^. Recommended rigid braces include: the Thuasne Eclipse™, which is adjustable and provides a solid chin rest; Aspen Vista™ with or without the thoracic extension for more support, or the Miami J™ for more specific fitting of long or short and thin necks and short stout necks. The Aspen is most commonly used but may cause temporomandibular joint pain in some patients; in patients with temporomandibular dysfunction, the Miami J brace is more forgiving to the mandible, and less likely to exacerbate temporomandibular pain. Soft neck braces may be better tolerated by some patients but must not encourage patients to adopt a forward head position.

Finally, patients in the High Irritability group might tolerate some proprioceptive and motor control training, focusing first on the pelvis and lumbar spine to provide a more stable base for the cervical spine. Patients with cervical instability should also work on lumbopelvic control, which will be variable in need and speed of progress, and in some may be necessary before commencing treatments for cervical motor control. All exercises need to be done correctly, engaging deep stabilizers/local muscles, while not over-recruiting global muscles. Exercises should be as functional as possible to ensure optimal motor patterning and retention, and to enhance carry-over into daily tasks. Adverse neural tension, motor control substitution strategies, etc., can lead to significant changes in the cervical spine area from movement elsewhere, even in the lower limbs. Some patients in the High Irritability group may also tolerate scapular motor control training and cautious cervical motor control. All motor control training should be performed with the spine in optimal alignment, which may require exercises typically done upright, to be done in supine or side lying instead. Any movement-based interventions should be discontinued if the patient appears to flare and postponed until they have progressed to a less irritable state. Table 11 also lists some interventions that should be avoided in this population, especially if the therapist is not a S-GJH/UCI expert.

Patients in the Moderate Irritability group should start with the interventions recommended in Table 11, though they may progress through these exercises more quickly than patients in the High Irritability group. Moderate Irritability patients can then progress to the interventions in Table 12. Functional training should address more demanding activities and positions (see Table 12). Patients need to be able to effectively grade their effort during exercises, and not use “all-or-none” patterns. Even when stronger and less symptomatic, it is good for patients to “warm up” by performing a few repetitions of the simpler, smaller exercises. This can help ensure optimal motor control, and allow patients to assess their status today using gentle exercises to ensure that they can tolerate their standard exercises on that day. Proprioception and motor control training can now typically involve the whole body to provide a more stable base for the cervical spine. This group may be able to progress to more cervical proprioceptive training using the head laser, beginning with static stabilization of the neck with arm or body movement, and only gradually progressing to small, controlled movement with the laser. More detailed examples of motor control training are provided in Supplementary Box 2. Patients may also tolerate training the cervical spine using the pressure biofeedback device. Table 13 lists interventions that may be appropriate for patients with Low Irritability, including higher level functional and motor control training, aerobic exercise, and more manual therapy to the cervical spine.

**TABLE 12:** Interventions for patients with moderate irritability.

● All interventions discussed in Table 11 Education ● Functional training, as described in Table 11 plus: Meal preparation, positional training for ADL and IADLs, standing, pivoting, stand pivoting, squatting, half-kneeling, pushing/pulling light objects, rotational upright core training, sweeping, shopping, light housework, carrying, driving, and lifting. Motor control and strength training ● Proprioception, motor control, and strengthening exercises for: ∘ Lower extremities, including knee, foot, ankle ∘ Shoulders and scapulae ∘ Thoracic spine ∘ Continue and progress for pelvis and lumbar spine ● Proprioception, motor control, and stabilization training for the cervical spine through available pain-free range. This may include using the head laser, starting by maintaining the head stable while moving the arms or legs, walking, and gradually progressing to small, controlled neck movements ● Gentle axial loading of the cervical spine (e.g., up to 1 pound/450 grams) if tolerated ● Low load cervical isometrics, with cuing to deactivate superficial muscles Manual therapy: ● Manual therapy for 1st rib, thoracic spine, acromioclavicular and sternoclavicular joints ● Soft tissue techniques for cervical muscles in spasm, physiological quieting ● Gentle manual techniques for C1 and C2 (if the therapist is trained) ● AVOID aggressive soft tissue or joint-based manual therapy to the cervical spine Aerobic exercise: ● E.g., Recumbent bike/peddler (if no neural tension signs); walking

**TABLE 13:** Interventions for patients with low irritability.

● All interventions discussed in Tables 11, 12 **Education** ● Functional training, as described in Tables 11, 12, plus: Occupation related functional training, i.e., prolonged desk work, phone, heavier household chores, gardening, etc. Sports specific training with precautions such as avoiding contact sports such as football or modification to sports such as no “heading” the ball **Manual therapy:** ● Additional muscle energy techniques in the cervical spine **Motor control and strength training** ● Proprioception and motor control using larger cervical ranges. Cervical axial loading may decrease symptoms during proprioceptive training ● Trunk-head coordination, eye-head coordination, eye-balance exercises ● Resistance training for the cervical spine ● Return to function/sport exercises, if appropriate, which may include more aggressive exercise, if tolerated. These may include perturbation, unpredictable challenges, and more endurance exercise for the neck **Aerobic exercise:** ● E.g., Walking, recumbent or upright bike. Some patients may tolerate running, swimming, aerobics with or without precautions

Case scenarios provided in Supplementary material demonstrate how the screening and assessment process guides intervention in patients with High, Moderate, and Low Irritability.

## Discussion

### The expert consensus process

These expert consensus recommendations fill a gap between the lack of research evidence supporting diagnosis and management of UCI in S-GJH and the wealth of expert clinical knowledge distributed across the globe. By involving 17 clinicians and researchers from 3 continents over a period of 1 year, we were able to elicit a wide range of perspectives and approaches. Having team leaders on each continent facilitate discussions with their groups and then present their team consensus to the full group, encouraged multiple perspectives and a rich discussion. This was a semi-structured approach, and should be followed up with both empirical research and rigorous consensus methods such as the Delphi method or Q methodology. While our large group size was an asset in providing a wealth of ideas and knowledge, the large group size significantly exceeded the maximum of seven participants recommended for nominal group method and had a broader charge than typically addressed using a nominal group method (41).

### The recommendations

The recommendations were intended to be accessible to physical therapists who were not experts in S-GJH or UCI. Recommendations were therefore relatively conservative to emphasize safety. None of the decisions discussed are absolute “black and white”; professional judgment is always essential. For example, RF signs might require urgent neurosurgical consultation or might only require monitoring. Some interventions might be safe in the hands of a PT knowledgeable about both S-GJH and UCI, but perhaps not as safe for a physical therapist less familiar with this population. The recommendations require professional judgment and therefore cannot be used as a recipe for evaluating and treating people with UCI. The High, Moderate, and Low Irritability cases presented in Supplementary material provide examples for how to use the flow chart model and recommendations.

The recommendations use S-GJH rather than G-HSD and hEDS because S-GJH is easily determined and does not require extensive G-HSD/hEDS knowledge. Allowing clinicians to use any hypermobility score encourages development and use of validated scores, while allowing for continued use of the well-known Beighton score, and the 5-Point Questionnaire for hypermobility when physical testing is not feasible (14, 17). If patients with S-GJH have not yet been diagnosed with G-HSD/hEDS, clinicians are encouraged to perform a more comprehensive patient assessment to identify other issues related to S-GJH. A recently described visual “Spider” web uses 25 validated questions to quantify the relative importance of eight different domains in which patients with G-HSD/hEDS often have problems: pain, fatigue, gastrointestinal, cardiac dysautonomia, urogenital, depression, and anxiety (31). The current recommendations included the validated “Spider” questions for depression and anxiety as one of the options for assessing YFs.

The current recommendations combine CCI and AAI into a single entity, UCI, because signs and symptoms overlap and it is not always possible to distinguish between CCI and AAI without specific imaging (5). However, CCI and AAI have different presentations and likely benefit from different management approaches. From an anatomical perspective, CCI is more likely to impact the lower cranial nerves, the long motor tracts, and the brainstem while AAI is more likely to impact the vertebrobasilar artery circulation, the occipital nerves and cause stretch injury to the upper spinal cord (1, 4, 7). CCI and AAI were combined into UCI for the purposes of these recommendations to avoid added complexity, and because many clinicians will not have access to diagnostic imaging studies to validate the distinction between CCI and AAI. Skilled physical therapists, however, may differentiate CCI and AAI, assess for anteroposterior versus rotational instability and manage these somewhat differently. Future work could add this element to the model.

These recommendations were developed specifically for patients with S-GJH; it is not clear whether they would also be applicable to patients with UCI due to other causes, such as rheumatoid arthritis (RA), trauma, etc. A recent publication suggested that EDS related cervical instability is substantially different from CCI due to RA and trauma, and that EDS-CCI is typically benign (2). However, instability in both conditions can result in myelopathy, cranial nerve neuropathy, brainstem compression, and vertebral artery injuries resulting in symptoms of neck pain, neck “clunking,” headache and facial pain (42, 43). In both conditions, identification of UCI can be challenging because signs and symptoms overlap with complex clinical presentations. Brainstem and vertebral artery compression can result in tinnitus, vertigo, visual disturbance, diplopia, dysphagia. Cranial nerve compression can result in dysphagia, dysarthria, loss of facial sensation, facial pain. Compression of the superior spinal cord and cervicomedullary junction can result in myelopathy, weakness, gait impairment, impaired dexterity, paresthesias, hyperreflexia, loss of abdominal reflex, Hoffman’s reflex, Babinski reflex, spasticity, loss of proprioception, bowel or bladder changes (7).

Most UCI tests for which sensitivity and specificity are known (13, 44), were deemed too provocative to be used in this population, especially since less provocative symptoms and tests were deemed sufficient. The recommendations differentiate signs and symptoms that are likely to be common but not diagnostic (sensitive) from those that are likely to be diagnostic but not always present (specific). Future research should assess the actual sensitivity and specificity of both signs and symptoms described in the current recommendation.

There are multiple comorbidities and differential diagnoses with signs and symptoms that overlap those of UCI (Table 10). Some may be more common in people with S-GJH due to excessive motion or abnormal tissue characteristics (e.g., Chiari malformation, dysfunctional myodural bridges, tethered cord) (45, 46). Prevalence of Low, Moderate, and High Irritability UCI is unknown. However, it is likely that many people with Low Irritability are undiagnosed and never receive physical therapy for this, or they receive appropriate physical therapy and resolve without complication. Patients with High Irritability are probably uncommon, except in specialty clinics. Hence, most patients receiving physical therapy start with Mild to Moderate Irritability and, when successfully managed, may have minimal signs and symptoms, but be vulnerable to recurrent flares.

### Limitations

This work has several limitations. It was not possible, within the scope of this paper, to define every clinical test and treatment approach. Therefore, these recommendations are intended for trained physical therapists familiar with standard physical therapy tests and interventions. The nature of the consensus group was such that there was a variety of expert experience and opinion, requiring considered facilitation to achieve consensus, especially regarding the structure of the model. However, the diversity of participants provided rich ideas, thoughtful critiques and, hopefully, a comprehensive perspective. We discovered both similarities and differences in how the international teams manage UCI. The creation of a simple model was challenging when most clinical decisions depend on multiple factors. This work could only touch on the depth of participants’ clinical experience managing this population. For example, participants wanted to include significantly more detail about motor control and proprioceptive assessment and training, autonomic nervous system balancing, and manual therapy approaches than space permitted. Future publications should hopefully build on this work.

### Future research

A multitude of research questions arise from these recommendations. These questions fall into two categories: those involving assessment and those involving management. Which of the symptoms and history (Table 1) are most sensitive and specific for UCI? What is the best way to assess irritability (Table 4)? Which RF signs and symptoms (Table 6) are most important? What are the sensitivity and specificity of the proposed tests (Tables 7–9)? What are the best ways to assess cervical proprioception and motor control in UCI? What patient subgroups are important to distinguish in UCI? For example, is it most important to differentiate between CCI and AAI, or anteroposterior vs. rotational instability? Is it important to differentiate among UCI affecting the spinal cord, medulla, cranial nerves or arteries/veins in managing UCI, or is management similar for all of these involved structures? Is UCI in S-GJH/hEDS different in important ways from UCI due to other conditions such as rheumatoid arthritis or trauma? What are the best ways to train proprioception and motor control anywhere in the kinetic chain? What manual techniques are safe and beneficial in UCI? What cervical braces and usage are most beneficial, and do these vary based on irritability? Hopefully, these questions will be addressed through systematic or scoping reviews or Delphi process research.

In conclusion, these recommendations provide an expert consensus model for describing, screening, performing physical examination and providing physical therapy for patients with S-GJH and UCI. The case scenarios in Supplementary material demonstrate how the recommendations might be used for a range of UCI irritability. The recommendations encourage identification of YF indicating a need for psychologically informed care or referral to another provider, as well as RF indicating a need for referral to an appropriate expert (e.g., neurosurgeon) either along with or instead of cautious conservative care. It benefits clinicians by providing safety recommendations for both physical examination and intervention, as well as treatment ideas. The recommendations can benefit patients through improved recognition of S-GJH/UCI, decreased likelihood of flares from the physical exam or intervention, and improved management. Participants in the consensus process agreed that most patients with S-GJH/UCI fall in the Low and Moderate Irritability groups and can do well with appropriate physical therapy. Though less common, patients with High Irritability are the most challenging to treat in physical therapy; nevertheless, education about body mechanics, functional training, and posture training are all likely to be beneficial even for patients who require surgery.

## Data availability statement

The original contributions presented in this study are included in the article/Supplementary material, further inquiries can be directed to the corresponding author.

## Ethics statement

Ethical review and approval was not required for the study on human participants in accordance with the local legislation and institutional requirements. Written informed consent from the patients/participants or patients/participants legal guardian/next of kin was not required to participate in this study in accordance with the national legislation and the institutional requirements.

## Author contributions

All authors contributed equally, with LR providing the initial idea, facilitating full group meetings, and facilitating the writing process. AH was a senior author, providing guidance, and support for the process.

## Footnotes

1 Dr. F. Henderson states: “It is imperative, however, that the patient perform isometric exercises four times a day for a total of 20 min, to prevent muscle wasting. Even gentle isometric resistance exercise in the brace will maintain muscle tone. Wearing the brace at night helps the patient to maintain alignment when most vulnerable to subluxing the neck–that is, when asleep. Moreover, realignment of the spine probably occurs with time in the brace.” (Personal communication, 10/1/22).
