# Supplementary material for: Presentation and physical therapy management of upper cervical instability in patients with symptomatic generalized joint hypermobility: International expert consensus recommendations
Source: Front Med (Lausanne). 2023 Jan 18;9:1072764. doi: 10.3389/fmed.2022.1072764 (PMC9893781; doi:10.3389/fmed.2022.1072764)
Supplement: Supplementary file 1 [file Presentation_1.pdf]

Russek LN, Block NP, Byrne E, Chalela S, Chan C, Comerford M, Frost N, Hennessy S, McCarthy A, Nicholson LL, Parry J, Simmonds J, Stott PJ, Thomas L, Treleaven J, Wagner W and Hakim A. (2022) Presentation and physical therapy management of upper cervical instability in patients with symptomatic generalized joint hypermobility: International expert consensus recommendations. Front. Med. 9:1072764. doi: 10.3389/fmed.2022.1072764

Supplementary Material: Case Scenarios

Patients described in these scenarios provided written informed consent. The cases include minor changes made to decrease the chance that subjects could be identified.

Case 1: High Irritability UCI (HI)

Demographics: Patient HI is a 26 y/o Caucasian female who had been employed as a hospital technician but is now unable to work.

Presenting problems & history, activity & participation restrictions: About 18 months ago, she began having severe migraines and pseudo-seizures, often triggered by lights, sounds and smells in the operating room at work. Episodes were severe enough 15 months ago that she went to the emergency room multiple times. She was let go from work, as she could not reliably complete her work shifts. Prior history: tonsilitis with tonsillectomy when young; episode of lockjaw; scoliosis; R sacroiliac joint pain; R shoulder labrum tear with surgical repair; gall bladder infection with removal. Starting 1 year ago, she developed widespread pain and re-injured her R shoulder (multidirectional instability). She was diagnosed with tethered cord and had surgical tethered cord release 10 months ago, which resolved low back tension that limited antigravity postures and decreased sacroiliac joint flares. However, her cervical instability issues seemed to get much worse after the tethered cord release. She has also been diagnosed with mast cell activation syndrome (MCAS), but it is not consistently symptomatic; she has multiple gastrointestinal problems, anaphylactic reactions to multiple medications, excessive swelling in response to bug bites, and hives when exposed to allergens. Gastrointestinal symptoms include pain, mixed irritable bowel syndrome, small intestinal bacterial overgrowth, and leaky gut. She also has postural orthostatic tachycardia syndrome (POTS) dominant dysautonomia.

Current symptoms include sensation of “bobble head”; heavy head; clunking and clicking in her neck; coat-hanger distribution pain with suboccipital HA; visual disturbances; tinnitus; dizziness; intermittent nausea; trouble swallowing, lump in throat; jaw pain; memory issues, brain fog, difficulty communicating. She reports experiencing “boat rocking” instability when standing; drop attacks (could be dysautonomia); pseudo-seizures; night sweats and poor temperature regulation. Rotating her head R triggers these symptoms and events; rotation L is painful but does not trigger neurological responses. Bright lights, sounds and smells can trigger neurological symptoms. Symptoms were also provoked by leaning forward, forward head posture, and sometimes by being upright with neck unsupported. Attempting to wear a hard cervical collar also exacerbates symptoms. Flares typically take more than 24 hours to resolve. In the past two months she reports recurrence of tethered cord symptoms: low back pain, symmetric lower extremity pain, restless legs, numbness on soles of feet, bowel/bladder dysfunction; lumbar MRI was negative for arachnoid adhesions.

Personal/Environmental factors: Her parents initially did not believe or understand why she was unable to work. Finances are tight, especially since she lost her job. She previously lived in an apartment with mold, which flared her symptoms; symptoms subsided slightly when she moved into a townhome, however her roommate does not respect her chemical and other sensitivities. Although there were no obvious Yellow Flags, her OSPRO-YF10[1] score of 39/53 indicated that there are, in fact, multiple Yellow Flags that need to be addressed.

Body Structure/Function Impairments: She is tall (5'9") and very thin, with a long, thin neck and long torso. Because of her High Irritability status, tests & measures focused on those from Table 7. Although she is sometimes able to ambulate without an assistive device, she uses a rollator walker or wheelchair when she has an 'attack' due to unsteadiness or for longer distances. LE demonstrate uncoordinated movement when walking. Posture demonstrates forward head and reversed cervical curve. Beighton score is 9/9. Speech is slurred, but no change in voice. Neurological testing: the uvula deviated R; Babinski, Hoffmann, and clonus are (+) bilaterally; deep tendon reflexes are hyper-reflexive. Other cranial nerve testing was negative. Eye gaze testing revealed difficulty with convergence, and this triggered a migraine with R sided facial, arm and leg weakness. Cervical range of motion was not formally assessed, but symptoms were triggered at 20º spontaneous rotation R or any flexion or extension.

Evaluation/Diagnosis/Prognosis: HI meets the criteria for S-GJH due to her Beighton score and symptoms. She presents with many symptoms consistent with both neurological and musculoskeletal UCI, and these symptoms are provoked by neck movement or postures that stress the neck (e.g., forward lean). She consistently meets all three criteria for High Irritability: her condition is severe, it is easily flared, and there is a prolonged time to calm after flare. Although she did not obviously present with Yellow Flags (YF), the OSPRO-YF10 demonstrated multiple YF that would benefit from being concurrently managed by a pain psychologist and psychologically informed physical therapy, as YF may exacerbate K's pain and disability and compromise her ability to actively engage in physical therapy.

She presents with many Red Flags (RF) signs and symptoms (Table 6), including: FASTER signs; pseudo-seizures; drop attacks; brain fog and intermittently altered consciousness; bowel/bladder changes; need to use a walker or wheelchair due to ataxia; abnormal central nervous system reflexes; some abnormal cranial nerve findings. Bowel/bladder changes might be attributed to possible re-tethering of her cord and drop attacks could be due to dysautonomia. Because the physical therapist is an S-GJH/UCI specialist, she chose to continue with a cautious examination and conservative management while referring to a neurosurgeon. Differential diagnoses to be considered include re-tethered cord, migraine, and dysautonomia unrelated to UCI. The patient was classified as High Irritability UCI with a strongly neurological presentation. Following the neurosurgical consultation, upright cervical flexion/extension MRI confirmed significant medullary kinking. EEG testing including shining a light in her R eye and flashing lights triggered her neurological symptoms and a pseudo-seizure.

---

[1] OSPRO-YF10 is the Optimal Screening for Prediction of Referral and Outcome Yellow Flags, 10 question version

Intervention: The patient was initially managed using all the patient education described in Table 11, as well as functional training (Box 1) to minimize flares during activities of daily living. She was given a hip/pelvic brace, knee braces, a sacral lock belt, and a posture support to promote stability throughout the body and assist with spinal alignment. She tolerated all the educational components well, with reports of improved sense of control and decreased anxiety about her condition. She did not tolerate a hard cervical collar as it provoked full body shaking and tremor within 1 minute of donning. Attempts to recruit lumbopelvic stabilizers in supine, attempting to perform a 10% activation Kegel with proprioceptive feedback resulted in full body shaking due to inability to coordinate muscle recruitment. In spite of several attempts, the patient was not able to do any lumbopelvic stabilization or diaphragmatic breathing without flares that would last several days. Since she could not tolerate active interventions recommended for High Irritability patients, it was not possible to progress physical therapy further.

Outcome and Follow-Up: Conservative care was provided 2x/week for 2 months while concurrently referring her for a neurosurgical consult. Although applying principles of self-care resulted in intermittent decrease in signs and symptoms, she was unable to progress to any movement training without flares. Her ataxic gait worsened until it was seldom safe for her to ambulate, even with a walker. At that time, she was referred back to the neurosurgeon, who is planning to do an upper cervical fusion (C0-C3).

Box 1

Examples of Functional Training for High Irritability Patients:

- Using a sitting hip hinge to bend forward for cleaning teeth, reaching at the table, leaning forward for eating foods, etc. In severely unstable UCI and intolerant to upright sitting wedge lying for eating. Use of a hard cervical collar to avoid excessive cervical-spine movement may be needed.
- Lying down: for putting in contacts
- Turning in bed: using one sided bridge and reach with top arm to log roll to side lying from supine lying
- Looking straight into the mirror for positional cueing: hair brushing, drying with blow dryer. Never bend into flexion/extension/side/rotate to dry hair. Use of a hard cervical collar to avoid excessive cervical spine movement may be needed.
- Belly button rule: Always face people you are talking with or directions you need to look. Cueing is your nose needs to stay in alignment with the belly button, turn your feet or body to keep alignment.
- Toileting: Avoid straining, use of squatty potty, long reacher for perineal care.
- Dressing: Dressing stick for donning shirts, lying down for pants, socks, slip on shoes or elastic shoelaces, long shoehorn, etc.
- Sexual activity (should be addressed): Person who has instability should be on the bottom position. The person without instability should be the more active partner. Clearance from MD is recommended.
- Cooking: Pre-packaged foods requiring little prep time, microwave meals, etc.
- Electronics usage: side-lying, supports for holding devices, holding phones at eye level, not looking down.

Case 2: Moderate Irritability UCI (MI)

Demographics: MI is a 43 year-old female presenting with widespread body pain with issues with her neck/jaw/headaches, wrist/hand, pelvis, and knee. She has been diagnosed with fibromyalgia, chronic pain, megacolon, and celiac disease. MI presented to physiotherapy to get a better understanding of her body for better functioning and ultimately less pain.

Presenting problems and key past history, activity/participation restrictions: MI presented with severe upper neck pain radiating into the lower neck and shoulders; daily intense headaches; vertigo; brain fog; blurred vision; arm weakness; pins and needles in arms; head heaviness/bobble head; clumsiness; and uncontrollable arm movements. Symptoms easily provoked by fast head movement; picking items off the floor; and car travel. Symptoms relieved with daily meditation; stretching; supine lying; and heat packs.

MI reported gastrointestinal distress with constipation since childhood; 5 repaired abdominal hernias (and over 20 unrepaired); fatigue; menstrual issues; vertigo; recurrent knee dislocations, and daily neck pain/headache. Knee dislocations began after childbirth.

Functionally MI is unable to work; feels socially isolated; has feelings of anxiety/depression; significant issues with housework; limited ability for walking/housework/cooking/dressing; unable to participate fully in the life of her young child; spends several days a week in bed to manage her symptoms.

Body Structure/Function Impairments: Standardized questionnaires/assessments were performed with of 4/5 on the 5-Point Questionnaire (for hypermobility); 6/9 Beighton score; Pain frequency (90%); intensity Scale (8/10); Patient Specific Functional Score; Neck Disability Index; Yellow flag screening from the Spider assessment tool; Pain Self Efficacy Questionaire (14/60); and Tampa Scale of Kinesiophobia (44%).

Evaluation/Diagnosis/Prognosis: Posture showed anterior pelvic translation; anterior pelvic tilt; upper abdominal muscles contracted at rest; flat thoracic spine; forward head translation; excessive upper cervical retraction; purposeful scapula retraction/depression (as instructed by prior health professionals).

MI showed bilateral poor hip control with one leg stance and single leg squat with positive Trendelenburg's and hip internal rotation with knee valgus position. Eye follow in sitting increased symptoms of vertigo/ visual disturbances.

Cervical range of motion was restricted to around one third of normal with excessive muscle gripping/guarding with extension being the most symptomatic with increased pain and vertigo with even a few degrees of chin lift. Cervical rotation range was increased significantly when both scapulae were repositioned by the therapist into an elevated position, indicating the muscles of this area are gripping to control neck motion. Full shoulder range of motion was present although excessive hitching of the scapulae increases pins and needles in arms as well as elicits coat hanger pain. Central nervous system testing was unremarkable.

Following the flow chart this client was classified as having Moderate Irritability UCI with the absence of any Red Flags. Assessment of cervical ligament stability was not attempted due to

the lack of it adding further to the clinical interpretations and the high likelihood of irritation of symptoms.

Hands-on assessment was conducted with the client in a supported supine position and intersegmental glides to reduce generalized muscle gripping (gentle muscle energy technique) were performed with good result on decreased dizziness, vertigo, pain, and increased freedom of cervical rotation (extension range unchanged).

Interventions: The clinical reasoning tool was used to assist with the selection of interventions that were likely to be tolerated by a patient with Moderate Irritability. If certain interventions were poorly tolerated a more cautious approach variation was included instead and vice versa.

Initial treatment of MI was focused on education about pacing strategies; relaxation of tension advised for upright postures; positioning to decrease neck muscle tension; and education for the client's self-care toolbox.

A soft neck collar was trialed and although feeling supportive and initially improving the amount of time upright, she was unable to tolerate it for longer periods as it placed her neck in a slightly more extended position than usual. Postural overcorrection and extreme tension in sitting and standing were addressed with MI to focus on elongation through the top of her head rather than chin tucking. This was very helpful and decreased her lower neck and shoulder blade pains.

A lower body and core stability program was commenced in a lying position with neck supported including diaphragmatic breathing exercises; isometric abdominal activations; and pelvic tilt. In supported sitting, postural work with a soft weight (300 grams) on her head for proprioceptive feedback was conducted.

Over subsequent sessions the use of a laser pointer mounted on a head strap was used to establish a "safe" zone of motion of the upper cervical spine and to demonstrate the proprioceptive control of her neck and head. Eye follow exercises (vertical, horizontal, and diagonal) in middle range of eye motion and with the neck supported were implemented to just elicit some feelings of vertigo that passed quickly once the eyes were closed for 2 seconds.

Lower body exercises were progressed to include squats and offset squats and shoulder strengthening with 0.5-1 kg hand weights. Isometric abdominal exercises in supine were progressed to include leg movements while maintaining pelvic neutral position. MI was able to incorporate the exercises into her already established daily stretching/meditation program and could exercise for 10-20 minutes per day.

Outcomes: MI has been formally diagnosed with hypermobile-Ehlers-Danlos syndrome by a rheumatologist; POTS (by a cardiologist); and is trialing new medications for POTS. MI's progress has included improved time upright; recovering from flares more quickly; decreased lower body pain; decreased frequency of knee dislocations. Her neck range has increased to around 66% of normal range but remains painful and tight although vertigo and neurological signs are lessened. Head extension control is still limited (to 33% of normal) and MI is reluctant to look up for more than a few minutes.

Ongoing plans including adding some light weight on her head to apply some gentle longitudinal proprioceptive input during neck and balance exercises. Specific strengthening of the deep neck flexors and deep cervical extensors will be introduced gradually once MI has recovered more confidence in her neck extension range in sitting (anxiety is due to her prior negative experiences with neck "strength exercises.")

<u>Case 3: Low Irritability UCI (LI)</u>

Demographics: LI is a 30 year-old single female with a long history of multiple musculoskeletal pain conditions. She has been an enthusiastic indoor rock climber, avid horse rider and competitive pole dancer and circus aerialist (Lyra & Silks). She is no longer a competitive performer although still works part-time as an aerial instructor. She has been unable to continue climbing but has recently returned to riding at low intensity. Her primary occupation requires sitting and prolonged telephone use. She now also receives government assistance to manage her medical conditions via the NDIS (Australia's National Disability Insurance Scheme). She maintains a reasonably active lifestyle.

Key presenting problems & history; activity & participation restrictions; personal factors: LI was diagnosed with hypermobile-Ehlers-Danlos syndrome (hEDS) 10 years ago and has subsequently been diagnosed with POTS, dysautonomia, chronic fatigue syndrome; and undiagnosed but probable MCAS. She maintains an active lifestyle which is intermittently derailed with POTS and dysautonomia and occasionally her musculoskeletal pain episodes. Her earlier musculoskeletal problems were primarily lumbo-pelvic (lumbar facet joint hyperextension impingement and sacroiliac joint pain and instability) with recurrent hamstring and adductor injuries along with neurodynamic sensitization. After a fall (landing on her right shoulder and head) 5 years ago, she started to develop significant neck pain, headaches and "brain fog". Her neck pain and migraine headaches have contributed to increasing disability and the need to transition away from aerial performance to more of a coaching/training role. She is also a keen horse rider, but her symptoms impact on her ability to maintain this activity.

Fatigue and pain limit many of her household, social, work, and recreational activities to short time frames of 10-15 minutes duration (F4 Functional Scale). The Neck Disability Index scores 33/50 indicating 'severe disability.' She has a Pain Self-Efficacy Questionnaire (PSEQ) score of 47/60. Scores above 40 are associated with return to work and maintenance of functional gains. The 'Yellow Flag' evaluation of the impact of anxiety and depression (validated for HSD) from the Spider Tool indicates only a mild impact on LI's daily life (though 18 months ago she experienced a period of significant depression).

Although LI has significant pain and disability, she has a good understanding of her condition and its myriad of contributing issues. She has a positive attitude towards her ability to use the many self-management tools she has available and has realistic expectations of her abilities and the challenges to maintain a healthy fulfilling lifestyle. Based on her history and subjective report, she was rated as Low Irritability, and all tests in Tables 7-9 were potentially appropriate, but still done cautiously.

Body Structure/Function Impairments: LI has a historical Beighton score of 9/9 with later development of thumb stiffness secondary to minor thumb injuries with aerial training. She is tall with a slim but muscular build and has soft, velvety skin with mild skin hyperextensibility and multiple locations of stria. She has a 'chin poke' upper cervical extension posture (common in aerialists) with a flat thoracic spine and downwardly rotated, winging scapula. She has hypermobile cervical flexion and rotation (active and passive rotation > 100° bilaterally). Cervical side bending is also hypermobile (ear to acromion). Cervical extension is also hypermobile but is limited by active protective guarding. She experiences upper cervical pain with headache referral while attempting to extend her head. She intermittently complains of dizziness/lightheadedness associated with extension activities and feels anxious that her "head is not stable" when she looks up. She uses active depression of the scapulae to generate passive tension in her scapula-cervical muscles and fascia to limit and control hypermobile range of motion.

Careful manual palpation demonstrates hypermobile C1-2 lateral flexion and C2 anterior translation with slight symptom provocation at end ROM. Passive hypermobile intersegmental translation in the upper cervical spine (C0-1-2), especially increased C2 lateral translation PAIVMs (passive accessory intervertebral movements) associated with side bending. She has mild to moderate alar ligament laxity on cautious manual assessment with minimal symptom provocation and no irritability (i.e., mild symptoms decrease immediately after testing). Upper limb neurodynamic testing is positive for median and suprascapular nerve loading. She finds both gentle cervical longitudinal distraction (head supported supine) relieving for her neck pain and gentle axial compression relieving for her headache symptoms (head supported supine). She also notes that sustained static head positions (e.g., working as relay officer) generates postural fatigue and can develop myofascial trigger point referral headaches as well.

Proprioceptively, joint position sense tested using a laser pointer is moderate to poor. She sees a neurologist for her migraines and in the past has had radiofrequency ablation of the lesser and greater occipital and auricular nerves with positive results. She has also had some Botox injections for her sternocleidomastoid muscles, with positive outcomes for her headaches.

Evaluation/Diagnosis/Prognosis: LI meets the criteria for hEDS and Low Irritability UCI without any YF or RF. She has hypermobile active cervical range of motion along with excessive accessory movement in the upper cervical spine. Alar ligament testing was deemed safe, though it should only be performed by experienced examiners and only when the patient fits the criteria for mild, non-irritable UCI. Cognitive movement control testing demonstrates inefficient coordination and poor functional control of the upper cervical spine during instruction to minimize/prevent upper cervical movement (especially extension and lateral flexion) while performing small range active movements of the scapulae, thoracic spine, or low cervical spine. Scapular postural control was inefficient with early fatigue associated with trying to maintain scapular upward rotation (poor recruitment efficiency and early fatigue of serratus anterior and middle/lower trapezius). Cervical muscle stabilizer motor control testing demonstrated inefficient recruitment and poor inner range activation of the deep cranio-cervical flexor and the oblique suboccipital extensor muscles. Her POTS and Dysautonomia frequently affects her ability to manage day to day activities and functional tolerance and needs to be more effectively managed.

Intervention: LI's management has prioritized improving cognitive control of upper cervical movement, with an emphasis on extension and lateral flexion control. A description of Graded Movement Control Training is given in Box 2, below. Retraining recruitment efficiency of the deep cranio-cervical flexor and oblique suboccipital extensor muscles to improve isometric and eccentric control of the hypermobile rotation and side bending was a secondary goal. Education regarding her hEDS, POTS and Dysautonomia and their influence on symptoms and functional limitations and strategies to control her UCI were also addressed. Over a timeframe of six months her rehab program incorporated cervical proprioceptive training using laser and other feedback exercises, and progressive movement control retraining. Graduated progressions involved adding light resistive loads to the upper extremity and adding small perturbation/displacement challenges to her neck. Further education included postural awareness observation, relaxation strategies and inhibitory stretching of tight, overrecruited mobilizer muscles, in particular the sternocleidomastoid and splenius capitis muscles. A consultant cardiologist and a neurologist continue to manage her POTS, Dysautonomia and migraine headaches.

Outcome and Follow-Up: Over a 6-month period she reported improved pain tolerance and postural endurance. She has had a significant decrease in the frequency and duration of the headaches, with only occasional migraines. She feels confident to move through the full (hypermobile) range of neck motion and has reduced anxiety and feeling of "instability" associated with extension movements of the head. She is still guarded and protective of sustained extension e.g., dentist, hairdresser; fast or sudden movements; or situations with the risk of unpredictable perturbations/displacement. She is still training other aerial performers (though no longer performing) and has stopped demonstrating techniques that wrap the head or neck or load the neck in any way. She has been able to return to horse riding (though not cantering or galloping) with care. LI has a battery of self-management tools to help her recover from intermittent flare ups more quickly. She has requested to maintain regular monthly clinical follow up sessions to maintain motivation and compliance and to build in progressions where appropriate.

Box 2. Principles & Strategies of Graded Movement Control Training for Upper Cervical Instability.

If there are non-provocative directions of neck movement and provocative directions of neck movement: - move away from provocation in the direction of relieving or non-provocative movement

↓

Cognitively control / prevent / limit / minimise movement in the upper cervical spine (palpation or visual feedback to maintain a neutral mid-range position), then move at an adjacent sites / region (e.g. lumbo-pelvic – thoracic spine – scapula – low cervical spine)

↓

Facilitate recruitment / activation of the Global Stabiliser (GS) muscles in the neck (lower Cx spine & upper cervical spine). Priority is given to GS muscles that can isometrically or eccentrically control the provocative / 'unstable' movements

A. Isometric activation in neutral positions

B. Small range concentric-eccentric movement towards shortened range (i.e. inner range hold & eccentric return)

C. Concentric activation through the full available inner range of motion and eccentric control through the non-provocative outer (often hypermobile) range of motion

↓

- Inhibit recruitment substitution & increase flexibility / extensibility of the over-active Global Mobiliser (GM) synergists

↓

- Add fatiguing resistance with a 'rotation challenge' (prioritise GS synergist) to increase strength & endurance efficiency
- Add a perturbation / unpredictable displacement challenge

If there are **NO** non-provocative directions of neck movement (i.e. all directions of neck movement are provocative) - consider whether this is due to:

a) nociplastic central sensitisation (clinical tip: symptoms do not respond to peripheral analgesics or NSAID medication) – Often requires central acting neuromodulator medication)

b) multidirectional uncontrolled translational movement (clinical tip: symptoms do respond well to peripheral analgesics or NSAID medication) – Requires prioritisation of Local Stabiliser (LS) muscle recruitment and retraining

↓

Facilitate recruitment / activation of the Local Stabiliser muscles in the neck (upper Cx spine +/- lower cervical spine) to control excessive / hypermobile intersegmental displacement / translation

- Cognitive isometric recruitment of LS synergists: Initially in a supported mid-range neutral position – with progression into recruitment in ,multiple different mid-range positions and then into unsupported postures

All cognitive recruitment and active movements are **initially performed with low / minimal contraction force (non-fatiguing)** and with **isometric recruitment or slowly through very small ranges of motion.** Progression into larger ranges of motion is only considered only after careful evaluation of tolerance
